# Reduced rates of primary joint replacement for osteoarthritis in Italian and Greek migrants to Australia: the Melbourne Collaborative Cohort Study

**DOI:** 10.1186/ar2721

**Published:** 2009-06-08

**Authors:** Yuanyuan Wang, Julie A Simpson, Anita E Wluka, Donna M Urquhart, Dallas R English, Graham G Giles, Stephen Graves, Flavia M Cicuttini

**Affiliations:** 1 Department of Epidemiology and Preventive Medicine, School of Public Health and Preventive Medicine, Monash University, Alfred Hospital, Commercial Road, Melbourne, VIC 3004, Australia; 2 Centre for Molecular, Environmental, Genetic and Analytic Epidemiology, School of Population Health, University of Melbourne, Swanston Street, Carlton, VIC 3053, Australia; 3 Cancer Epidemiology Centre, The Cancer Council Victoria, Rathdowne Street, Carlton, VIC 3053, Australia; 4 Baker Heart and Diabetes Research Institute, Commercial Road, Melbourne, VIC 3004, Australia; 5 Department of Orthopaedic Surgery, University of Melbourne, Royal Melbourne Hospital, Gratten Street, Parkville, VIC 3050, Australia; 6 AOA National Joint Replacement Registry, Discipline of Public Health, School of Population Health & Clinical Practice, University of Adelaide, North Terrace, Adelaide, SA 5005, Australia

## Abstract

**Introduction:**

Racial and ethnic disparities in rates of total joint replacement have been described, but little work has been done in well-established migrant groups. The aim of this study was to compare the rates of primary joint replacement for osteoarthritis for Italian and Greek migrants to Australia and Australian-born individuals.

**Methods:**

Eligible participants (n = 39,023) aged 27 to 75 years, born in Italy, Greece, Australia and the United Kingdom, were recruited for the Melbourne Collaborative Cohort Study between 1990 and 1994. Primary hip and knee replacement for osteoarthritis between 2001 and 2005 was determined by data linkage to the Australian Orthopaedic Association National Joint Replacement Registry.

**Results:**

Participants born in Italy and Greece had a lower rate of primary joint replacement compared with those born in Australia (hazard ratio [HR] 0.32, 95% confidence interval [CI] 0.26 to 0.39, *P *< 0.001), independent of age, gender, body mass index, education level, and physical functioning. This lower rate was observed for joint replacements performed in private hospitals (HR 0.17, 95% CI 0.13 to 0.23), but not for joint replacements performed in public hospitals (HR 0.96, 95% CI 0.72 to 1.29).

**Conclusions:**

People born in Italy and Greece had a lower rate of primary joint replacement for osteoarthritis in this cohort study compared with Australian-born people, which could not simply be explained by factors such as education level, physical functioning, and weight. Although differential access to health care found in the population may explain the different rates of joint replacement, it may be that social factors and preferences regarding treatment or different rates of progression to end-stage osteoarthritis in this population are important to ethnic disparity.

## Introduction

Total joint replacement has been recognized as a highly efficacious and cost-effective procedure for the treatment of advanced hip and knee osteoarthritis (OA) in its capability to relieve pain, increase mobility, and improve the quality of life [1-3]. The majority of knee and hip replacements are performed for OA [4]. Since OA is not a life-threatening disease, total joint replacement is an elective option available to patients for the purpose of improving their quality of life.

The racial and ethnic disparities in the rates of total joint replacement have been well documented in the US, where African-Americans and Hispanics have substantially lower rates of hip and knee joint replacement compared with Caucasians [5-10]. The origins of these disparities are complex. The differences in the rates of total hip and knee replacement cannot be attributed to differences in the prevalence of OA since there is evidence that the prevalence of OA is similar among these ethnic groups [11,12]. Potential sources of the disparities may include access to health care, physician bias, patient-physician communication, and patient-level factors [13-18]. Studies on the ethnic disparity of joint replacement other than in the US population are limited. Although the prevalence of symptomatic hip and knee OA in Italy and Greece is similar to that of other countries [19], there are some data to suggest that Italian people in Italy have a low rate of joint replacements [20].

Overseas migration has played a key role in shaping Australia as one of the most culturally diverse nations in the world. According to preliminary estimates for 2005, 24% of the Australian population was born overseas [21]. After migrants from the UK and New Zealand, those from Italy and Greece are among the most common migrant groups [21] and accounted for 1.1% and 0.6%, respectively, of the total Australian population in the 2005 census [22]. Italian and Greek immigration increased dramatically after World War II. Italian and Greek migrants arrived at Australia in the largest numbers in the decades immediately following. World War II. They were among the main groups targeted by Australian Government Migration Schemes in the 1950s and 1960s to deal with labour shortages in Australia. To be accepted, individuals needed to be of European ancestry, reasonably healthy, and without a criminal record. Most of the immigrants who arrived in Australia in those decades were unskilled and had little or no formal education, and only a minority had higher levels of education. Most migrants arriving in the 1950s were in their twenties. Thus, people coming from Italy and Greece represent the older migrant streams [22].

The aims of this study were to use the Melbourne Collaborative Cohort Study (MCCS) to examine whether Australian-born people and migrants to Australia from Italy or Greece had different rates of primary joint replacement performed for OA and to determine whether any differences could be accounted for by socioeconomic factors and known risk factors for OA. We hypothesize that Italian and Greek migrants to Australia have a lower rate of joint replacement compared with Australian-born individuals and that this discrepancy is not explained by differences in risk factors for OA or in education.

## Materials and methods

### The cohort

The MCCS is a prospective cohort study of 41,528 residents (17,049 men) of Victoria, Australia, between 27 and 75 years of age at baseline, 99.3% of whom were 40 to 69 years of age [23]. Participants were recruited via electoral rolls (registration to vote is compulsory for Australian adults), advertisements, and community announcements in the local media (for example, television, radio, and newspapers) between 1990 and 1994. Southern European migrants to Australia (including 5,425 from Italy and 4,535 from Greece) were deliberately oversampled to extend the range of lifestyle exposures and to increase genetic variation. The study protocol was approved by the Human Research Ethics Committee of The Cancer Council Victoria. Follow-up was conducted by record linkage to electoral rolls, electronic phone books, the Victorian Cancer Registry, and death records. To update lifestyle exposures, the cohort was followed up with by mailed questionnaire and (as necessary) by telephone from 1995 to 1998 (first follow-up) and by face-to-face interviews from 2003 to 2007 (second follow-up).

### Melbourne Collaborative Cohort Study data

#### Demographic and anthropometric data

Extensive information was collected at baseline (1990 to 1994) in face-to-face interviews that included questionnaires and physical measurements [23]. Demographic data, including date of birth, country of birth, and education level, were collected via questionnaire. Physical measurements, including height and weight, were directly measured using standardized written protocols [24]. Body mass index (BMI) (kilograms per square metres) was calculated as weight (kilograms) divided by the square of height (metres).

#### Physical functioning and self description of health status

At the first follow-up of the MCCS, physical functioning was assessed by asking five questions: Did health problems limit you in your everyday physical activity? Did pain interfere with your normal work? Has your physical health or emotional problems interfered with your normal social activities? Have you been bothered by emotional problems? Was it difficult doing your daily work because of your physical health or emotional problems? Self description of health status was assessed by asking: In general, how would you describe your health?

#### Self-reported joint replacement

From 2003 onward, 28,046 study participants (68% of the original MCCS participants) took part in the second follow-up. The participants were asked questions about their first joint replacement surgery: Have you ever had a hip replacement? When did you have your first hip replacement? Have you ever had a knee replacement? When did you have your first knee replacement?

### Study participants

Of the 41,528 participants recruited, 2,505 (6.0%) were excluded from analysis because they died or left Australia prior to 1 January 2001 (n = 1,758), or had undergone a sex change since baseline (n = 2), or had reported a primary joint replacement prior to 1 January 2001 at the second follow-up of the MCCS (n = 631) or their first recorded procedure was a revision joint replacement as recorded in the Australian Orthopaedic Association National Joint Replacement Registry (AOA NJRR) (n = 114), thus leaving 39,023 participants eligible for analysis.

### Identification of incident primary knee and hip joint replacement

All participants gave written consent allowing access to their medical records. Cases were identified from the AOA NJRR. The AOA NJRR commenced in 1999 and was introduced in a staged state-by-state approach that was completed nationally by mid-2002. Victorian data collection commenced in 2001. The registry monitors the performance and outcome of both hip and knee replacement surgery in Australia. It has detailed information on the prostheses and surgical technique used and the clinical situation used for both primary and revision joint replacement [25]. By using detailed matching technology, it is able to determine the success (or lack thereof) of the joint replacement surgery. Although data collection for the registry is voluntary, it receives cooperation from all hospitals undertaking joint replacement surgery [25].

The NJRR validates its data by using both internal systems and external data sources. The most important external data source is state health department data. Validation of registry data against health department recorded data involves a sequential multilevel matching process. Following the validation process and the retrieval of unreported records, the registry collects the most complete set of data relating to hip and knee replacement in Australia [4].

Identifying information of MCCS participants, including first name, last name, date of birth, and gender, was provided to the AOA NJRR in order to identify those MCCS participants who had had a primary or revision joint replacement between 1 January 2001, when the registry began to collect Victorian data, and 31 December 2005. The matching was performed on these data provided using US Bureau of the Census Record Linkage Software. Exact matches were identified and probabilistic matches were reviewed. Among the 1,380 MCCS participants (corresponding to 1,655 NJRR procedures) identified, 1,360 (98.6%) were exact matches. One hundred eighty-five participants were matched on date of birth, and 47 were matched on first name and last name. Information on patient address was then used to investigate the possible matches. The study was approved by the Human Research Ethics Committee of The Cancer Council Victoria and the Standing Committee on Ethics in Research Involving Humans of Monash University.

### Statistical analysis

Follow-up for primary joint replacement (that is, calculation of person-time) began on 1 January 2001 and ended on the date of first primary joint replacement for OA or the date of censoring. Subjects were censored at the date of first primary joint replacement performed for indications other than OA, the date of death, the date they left Australia, or the end of follow-up (that is, 31 December 2005, when ascertainment of joint replacement by NJRR was complete), whichever came first.

The exposures of interest were country of birth (Australia, the UK, Italy, and Greece), age, gender, BMI, education level (either primary and some secondary or completed secondary and degree/diploma), and physical functioning. For the five individual physical functioning questions (scored from 1 for 'not at all' to 5 for 'extremely'), reliability analysis showed a Cronbach alpha coefficient of 0.86, which indicated a good internal consistency of these questions. Thus, the scores were added to obtain a combined score of physical functioning for each individual (ranging from 5 to 25). The physical functioning limitation was then collapsed into four categories: none (score 5), mild (score 6–10), moderate (score 11–15), and severe (score 16–25).

Cox proportional hazards regression models were used to estimate the hazard ratios (HRs) of primary joint replacement for OA associated with each of the above exposures. To estimate HRs separately for the risk of joint replacement undertaken in private and public hospitals associated with different country of birth and to test for heterogeneity, Cox models based on competing risks were fitted using a data duplication method [26].

Tests based on Schoenfeld residuals and graphical methods using Kaplan-Meier curves showed no evidence that proportional hazard assumptions were violated for any of the exposures. A *P *value of less than 0.05 (two-sided) was considered statistically significant. All statistical analyses were performed using Stata (Intercooled Stata 9.2 for Windows; StataCorp LP, College Station, TX, USA).

## Results

### Descriptive characteristics of study population

A total of 1,009 primary joint replacements (541 knee replacements and 468 hip replacements) performed for OA were identified between 1 January 2001 and 31 December 2005. Descriptive statistics of the study participants are shown in Table 1. The participants born in Italy and Greece were less likely to be women and had higher BMI, lower education, lower self description of health status, and more severe physical functioning limitation when compared with those born in Australia. Participants born in the UK had characteristics similar to those of Australian-born participants except that the former were less likely to be women.

**Table 1:** Characteristics of study population by country of birth

	Italy-born n = 5,083	Greece-born n = 4,304	Australia-born n = 26,789	UK-born n = 2,847
Age when entering MCCS, years	56.2 ± 7.9	55.0 ± 7.6	54.7 ± 8.9	54.7 ± 8.5
Age when entering JR cohort, years	64.8 ± 8.1	63.5 ± 7.7	62.3 ± 9.1	61.6 ± 8.5
Women^a^	2,888 (56.8)	2,381 (55.3)	16,518 (61.7)	1,539 (54.1)
Body mass index, kg/m^2^	28.9 ± 4.3	28.9 ± 4.1	26.2 ± 4.3	26.4 ± 4.0
Education^a^				
Primary and some secondary	4,218 (84.8)	3,575 (85.4)	13,174 (49.2)	1,144 (40.3)
Completed secondary and degree/diploma	756 (15.2)	609 (14.6)	13,604 (50.8)	1,694 (59.7)
Self description of health status^a^				
Poor/Fair				
Good/Very good/Excellent	1,211 (33.1) 2,444 (66.9)	837 (27.4) 2,214 (72.6)	2,960 (12.4) 20,929 (87.6)	333 (13.4) 2,151 (86.6)
Physical function limitation^a^				
None (score = 5)	1,137 (31.8)	1,221 (41.0)	6,997 (29.9)	748 (30.8)
Mild (score 6–10)	1,659 (46.3)	1,073 (36.0)	11,825 (50.5)	1,221 (50.2)
Moderate (score 11–15)	551 (15.4)	416 (14.0)	2,963 (12.7)	303 (12.5)
Severe (score 16–25)	232 (6.5)	268 (9.0)	1,610 (6.9)	159 (6.5)
Any primary JR^a^	84 (1.7)	40 (0.9)	812 (3.0)	73 (2.6)
JR in private hospitals^a^	42 (0.8)	10 (0.2)	658 (2.4)	50 (1.8)
JR in public hospitals^a^	42 (0.8)	30 (0.7)	154 (0.6)	23 (0.8)
Primary hip JR^a^	39 (0.8)	14 (0.3)	383 (1.4)	32 (1.1)
Primary knee JR^a^	45 (0.9)	26 (0.6)	429 (1.6)	41 (1.4)

Values are reported as mean ± standard deviation or as ^a^number (percentage). JR, joint replacement; MCCS, Melbourne Collaborative Cohort Study.

The MCCS cohort had a reduced rate of primary joint replacement compared with the population from Victoria (the Australian state from which the MCCS cohort was recruited) over this time period. For knee and hip joint replacement restricted to those 55 to 84 years old, the standardized incidence ratio was 0.80 (95% confidence interval [CI] 0.76 to 0.85). However, the standardized incidence ratios were 0.96 (95% CI 0.90 to 1.02) for those born in Australia or the UK and 0.38 (95% CI 0.32 to 0.45) for those born in Italy or Greece when compared with the Victorian population.

### Incidence rates of primary joint replacement for osteoarthritis

The incidence rate of primary joint replacement for OA was 5.4 (95% CI 5.0 to 5.7) per 1,000 person-years for the whole study population. Participants born in Italy or Greece had a reduced incidence rate of primary joint replacement compared with those born in Australia or the UK (2.7 [95% CI 2.3 to 3.2] versus 6.2 [95% CI 5.8 to 6.6] per 1,000 person-years, *P *< 0.001). After adjustments for age, gender, BMI, and education level were made, Italian- and Greek-born participants had decreased rates of primary joint replacement (HR 0.39 [95% CI 0.31 to 0.49] and 0.24 [95% CI 0.17 to 0.33], respectively, all *P *< 0.001) for both primary hip and knee replacement when compared with Australian-born participants. In contrast, participants born in the UK had a similar rate of joint replacement compared with Australian-born people (HR 0.90 [95% CI 0.71 to 1.15], *P *= 0.40) (Table 2). Self description of health status was lower and physical functioning limitation was more severe for those born in Italy or Greece (Table 1), and adding these variables to the regression model had little effect on the HR for primary joint replacement (HR 0.36 [95% CI 0.29 to 0.46], *P *< 0.001).

**Table 2:** Risk factors for primary joint (hip and knee) replacement for osteoarthritis

	Primary joint replacement		Primary hip replacement		Primary knee replacement	
			
	Hazard ratio (95% CI)	*P *value	Hazard ratio (95% CI)	*P *value	Hazard ratio (95% CI)	*P *value
Country of birth						
Australia	1.00 (reference)	-	1.00 (reference)	-	1.00 (reference)	-
UK	0.90 (0.71–1.15)	0.40	0.81 (0.57–1.17)	0.26	0.99 (0.72–1.36)	0.95
Italy	0.39 (0.31–0.49)	< 0.001	0.45 (0.31–0.63)	< 0.001	0.35 (0.25–0.48)	< 0.001
Greece	0.24 (0.17–0.33)	< 0.001	0.22 (0.13–0.38)	< 0.001	0.25 (0.17–0.38)	< 0.001
Italy/Greece combined	0.32 (0.26–0.39)	< 0.001	0.35 (0.26–0.47)	< 0.001	0.31 (0.24–0.40)	< 0.001
Age per 1 year	1.08 (1.07–1.09)	< 0.001	1.07 (1.06–1.09)	< 0.001	1.08 (1.07–1.10)	< 0.001
Body mass index per 1 kg/m^2^	1.10 (1.09–1.11)	< 0.001	1.05 (1.03–1.07)	< 0.001	1.13 (1.12–1.15)	< 0.001
Gender (female vs. male)	1.08 (0.95–1.23)	0.25	1.06 (0.88–1.28)	0.55	1.08 (0.90–1.29)	0.42
Education						
Primary and some secondary	1.00 (reference)	-	1.00 (reference)	-	1.00 (reference)	-
Completed secondary and degree/diploma	1.15 (1.01–1.32)	0.04	1.37 (1.13–1.66)	0.001	0.98 (0.81–1.18)	0.81

Values are mutually adjusted for age, gender, body mass index, country of birth, and education level. CI, confidence interval.

When the incidence rates of primary joint replacement performed in private and public hospitals were examined separately, Italian- and Greek-born participants had a reduced incidence rate of primary joint replacement undertaken in private hospitals (1.1 [95% CI 0.9 to 1.5] versus 5.0 [95% CI 4.6 to 5.3] per 1,000 person-years, *P *< 0.001), but a similar rate of primary joint replacement undertaken in public hospitals (1.6 [95% CI 1.3 to 2.0] versus 1.2 [95% CI 1.1 to 1.4] per 1,000 person-years, *P *= 0.09), when compared with those born in Australia or the UK. After adjustments for age, gender, BMI, and education level were made, participants born in Italy or Greece had a decreased rate of joint replacement undertaken in private hospitals compared with those born in Australia (HR 0.24 [95% CI 0.18 to 0.33] and 0.08 [95% CI 0.04 to 0.14], respectively, all *P *< 0.001), but a similar rate of joint replacement undertaken in public hospitals (Table 3).

**Table 3:** Relationship between country of birth and rates of primary joint replacement in private and public hospitals

	Primary joint replacement in private hospitals (n = 760)		Primary joint replacement in public hospitals (n = 249)		Heterogeneity of hazard ratios^a^
			
	Incidence rate (95% CI)^b^	Hazard ratio (95% CI)^a^	Incidence rate (95% CI)^b^	Hazard ratio (95% CI)^a^	*P *value
Australia	5.1 (4.7–5.5)	1.00 (reference)	1.2 (1.0–1.4)	1.00 (reference)	-
UK	3.6 (2.8–4.8)	0.76 (0.57–1.01)	1.7 (1.1–2.5)	1.50 (0.97–2.32)	0.01
Italy	1.7 (1.3–2.3)	0.24 (0.18–0.33)^c^	1.7 (1.3–2.3)	0.99 (0.69–1.41)	< 0.0001
Greece	0.5 (0.3–0.9)	0.08 (0.04–0.14)^c^	1.4 (1.0–2.1)	0.92 (0.61–1.39)	< 0.0001
Italy/Greece combined	1.1 (0.9–1.5)	0.17 (0.13–0.23)^c^	1.6 (1.3–2.0)	0.96 (0.72–1.29)	< 0.0001

^a^Adjusted for age, gender, body mass index, and education level. ^b^Per 1,000 person-years. ^c^Statistically significant, *P *< 0.001. CI, confidence interval.

## Discussion

In this prospective cohort study, participants born in Italy or Greece had a significantly reduced rate of primary joint replacement for OA compared with Australian- or UK-born individuals, which was independent of age, gender, BMI, education level, self description of health status, and physical functioning. This was consistent when primary hip and knee replacements were analysed separately. Moreover, the reduced rate was observed for joint replacements performed in private hospitals, but not for joint replacements performed in public hospitals.

The racial and ethnic disparity in the rates of joint replacement has been well documented in the US. Although the disparity is generally thought to be largely due to lack of access and to social disadvantage, factors such as treatment preference, patient perception, and sociocultural beliefs may also contribute [13-18]. For example, Caucasians are more likely to undergo total knee and hip replacement compared with African-Americans [5-10]. Total knee replacement rates among Hispanics are higher than among African-Americans but are lower than among Caucasians [7]. Hispanics with Medicare receive total hip replacement at lower rates than non-Hispanics [27]. We also observed ethnic disparity in joint replacement rates in this cohort study. Although the prevalence of symptomatic hip and knee OA in Italian and Greek people is similar to that of other populations [19], we found a significantly reduced rate of primary joint replacement among those born in Italy and Greece compared with those born in Australia, independent of age, gender, BMI, and educational level.

There are a number of possible explanations for the lower rate of joint replacement for migrants from Italy and Greece. In our study, Italian and Greek migrants had more severe physical function limitation and worse self-described health status in comparison with Australian-born people, suggesting that the former may have higher or at least similar levels of need. However, they had lower rates of joint replacement than Australian-born people. It is possible that the observed differences were a result of inequalities in access to health care. This is supported by the difference in the rates of joint replacement performed in private hospitals for those born in Italy or Greece, each of which requires the patient to have private health insurance or very substantial 'out of pocket' costs for this procedure. This was more evident in the Greek migrants, who had an HR of 0.08 for joint replacement performed in private hospitals but an HR of 0.92 for joint replacement performed in public hospitals when compared with Australian-born participants. Australia has a publicly funded universal health insurance system (Medicare) and people who do not have private health insurance have access to quality health care service under this system. If the need for joint replacement were similar between Italian and Greek migrants and Australian-born people and if differential access to health care were the only explanation for the lower rate of joint replacement in Italian and Greek migrants, we would expect that any unmet need may have been reflected in a disproportionate increase in the rate of joint replacement in the public hospital system for this group. However, this was not observed in our study; the standardized incidence ratio in those born in Italy and Greece was very low at 0.38 (95% CI 0.32 to 0.45) and they had a similar rate of joint replacement performed in public hospitals compared with Australian-born people. It does raise issues of whether there are other patient-related factors that affect the utilization of joint replacement in those with an Italian or Greek background.

Another possible explanation for this ethnic disparity may relate to health beliefs and preferences for treatment [15-18,28]. A recent study showed that there were significant differences in health-related beliefs, in particular in relation to reduced perceived benefits of total joint replacements, and more perceived barriers to total joint replacement for African-Americans compared with Caucasians [29]. There are no data available as to whether such barriers might exist in those of Italian or Greek background and thus result in a reduced access to joint replacement surgery. In addition, it may be that differences in family support arrangements enable Italian and Greek migrants to cope better with significant OA and either avoid or delay the onset of joint replacement. We were unable to examine these factors in this study. A language barrier for Italian and Greek migrants may also provide a possible explanation for the observed ethnic disparity for joint replacement. There is evidence that language is a common barrier in health care settings, affecting medical comprehension and increasing the risk of adverse medication reactions [30-32]. Breaking the language barrier is the critical first step to reduce health care disparities [33]. It is likely that, due to a language barrier, Italian and Greek migrants lack good communication with health care providers, are less familiar with joint replacement surgery, or have greater perception of risk and thus would not prefer joint replacement as a treatment for severe OA.

A further potential explanation is that, although the OA prevalence in Italian and Greek people is similar to that in other populations [19], it is possible that those born in Australia or the UK have a higher rate of more severe or end-stage OA requiring a joint replacement. It is possible that genetic or environmental factors (such as diet) of the migrants from Italy and Greece may confer a protective effect on the progression of hip and knee OA, despite their relative obesity. For example, the notion of the beneficial effects of the Mediterranean diet is well described in the area of cardiovascular disease [34,35] and recent work has suggested that diet, in particular increased vitamin C and reduced fatty acids, has a beneficial effect on joint health [36,37].

Strengths of our study include the large sample size and prospective study design. Our results are further strengthened by the prospectively collected demographic data and the directly measured height and weight, which are more reliable than self-reported data. However, there are a number of potential limitations in this study. There may be a selection bias. The MCCS is a healthy volunteer cohort with lower rates of mortality, cardiovascular disease, and cancer compared with the general population [23]. The participants are likely to be more health conscious than the general population, as in most voluntary cohort studies. Migrants to Australia from Italy and Greece were deliberately oversampled to extend the range of lifestyle exposures and to increase genetic variation, making the MCCS a heterogeneous cohort. Greek and Italian migrants are likely to be different from the home population in terms of health status and socioeconomic status. In particular, there is likely to be a 'healthy migrant effect' resulting from a self-selection process that includes people who are willing to migrate and excludes those who are sick or disabled. Most of the immigrants who arrived in Australia during the decades after World War II were unskilled and had little or no formal education, and only a minority had higher levels of education. However, representativeness is not necessary for the estimation of associations between exposures and subsequent health outcomes with a high degree of internal validity, but we cannot use the MCCS to derive population estimates of disease prevalence and incidence. There is no selection bias in terms of ascertainment of joint replacement since the identification of joint replacement is based on linkage to the AOA NJRR, which has very comprehensive coverage in Australia.

The recruitment of MCCS participants and data collection commenced in 1990 to 1994. The NJRR started joint replacement data collection in Victoria in 2001. Thus, we do not have complete and reliable joint replacement data for the study population prior to 2001. Although we excluded those MCCS participants who reported a joint replacement prior to 2001 at the second follow-up, this information may be unreliable and is known for only 68% of the original cohort. As a result, some misclassification of joint replacement status may have occurred, although it is likely to have been nondifferential in relation to the studied risk factors, subsequently underestimating the strength of any observed associations. In addition, participants born in Australia or the UK had a joint replacement incidence similar to the general Australian population over the same time period.

## Conclusions

People born in Italy or Greece had a lower rate of primary joint replacement in this cohort study compared with those born in Australia, and this difference could not be explained merely by factors such as education level, physical functioning, and weight. Although access to health care may play a role, it may be that social factors and preferences regarding treatment or different rates of progression to end-stage OA in this population are important. This warrants further investigation since it is unclear whether efforts aimed at education regarding potential benefits of joint replacement are needed to deal with this difference, or alternatively, it may be that genetic or lifestyle factors in the Italian or Greek population identify novel factors that prevent progression to end-stage OA despite their relative obesity.

## Abbreviations

AOA: Australian Orthopaedic Association; BMI: body mass index; CI: confidence interval; HR: hazard ratio; MCCS: Melbourne Collaborative Cohort Study; NJRR: National Joint Replacement Registry; OA: osteoarthritis.

## Competing interests

The authors declare that they have no competing interests.

## Authors' contributions

YW participated in the design of the study, performed the statistical analysis and the interpretation of data, and drafted the manuscript. JAS participated in the acquisition of data, helped to perform the statistical analysis, and reviewed the manuscript. AEW and DMU helped in the interpretation of data and reviewed the manuscript. DRE and GGG participated in the design of the study and the acquisition of data and reviewed the manuscript. SG participated in the design of the study and the acquisition of data, helped in the interpretation of data, and reviewed the manuscript. FMC participated in the design of the study, helped in the interpretation of data, and reviewed the manuscript. All authors read and approved the final manuscript.
